# Sensory feedback for gait in transtibial amputees: a narrative review of technologies and clinical outcomes

**DOI:** 10.3389/fbioe.2025.1722817

**Published:** 2026-01-05

**Authors:** Rita Suliman Hussain, Nasrul Anuar Abd Razak, Mahmoud Alfatafta, Chan Chow Khuen, Bashar Al Qaroot, Timothé Ramousse

**Affiliations:** 1 Department of Biomedical Engineering, University Malaya, Kuala Lumpur, Malaysia; 2 Department of Physics, Faculty of Science and Technology, Airlangga University, Surabaya, Indonesia; 3 Department of Prosthetics and Orthotics, School of Rehabilitation Sciences, The University of Jordan, Amman, Jordan; 4 Department of Biomedical Engineering, School of Polytech Marseille, Aix-Marseille University, Marseille, France

**Keywords:** transtibial amputation, somatosensory feedback, vibrotactile stimulation, electrotactile stimulation, gait symmetry, rehabilitation engineering

## Abstract

The loss of somatosensory feedback in transtibial amputees contributes to gait asymmetry, increased metabolic cost, higher fall risk, and dependence on visual cues. Sensory neuroprostheses, both invasive and non-invasive, provide methods to restore aspects of this feedback and enhance functional mobility. This review combines recent findings from invasive procedures, such as intraneural electrodes, spinal cord stimulation, and agonist–antagonist myoneural constructs, with non-invasive electrotactile, mechanotactile, and vibrotactile techniques. Invasive systems offer high-quality somatotopic mapping that improves embodiment and reduces phantom limb pain but are limited by surgical risks, long-term stability issues, and scalability concerns. Non-invasive systems, especially those utilizing vibrotactile feedback, have demonstrated measurable benefits, including a 41% increase in walking speed, fewer stumbles, improved symmetry indices from approximately 60% to roughly 75%, and a 70% reduction in phantom limb pain in small clinical trials. However, most studies are limited by small sample sizes, short intervention durations, and laboratory-based testing, with inconsistent reporting of cognitive load, training doses, and real-world applicability. The analysis here highlights vibrotactile systems as the most practical near-term option for clinical use, while invasive approaches remain valuable for high-performance tasks requiring precise feedback. Future research should focus on larger, longer trials, standardized outcome reporting, and seamless integration of sensors and actuators into prosthetic sockets. Overcoming these challenges is key to developing scalable, next-generation sensory feedback systems capable of restoring natural and confident gait in transtibial amputees.

## 1 Introduction

Proprioception is the body’s intrinsic ability to sense the position, movement, and force of its limbs without visual input (Marasco and De Nooij, 2022). It relies on mechanoreceptors in muscles, tendons, joints, and deep tissues that send information about joint orientation, muscle stretch, and load to the central nervous system (CNS) (Proske and Gandevia, 2012).

Proprioception functions at both conscious and unconscious levels (Gelener et al., 2021), consisting of two main components: static limb position, which indicates body segment orientation, and kinesthesia, which involves the perception of movement and speed (Johnson et al., 2008). This internal sensory feedback is crucial for motor control and balance, enabling coordinated actions such as walking, reaching, and maintaining stability (Santuz et al., 2022; Alonso et al., 2023).

Although proprioception is part of the broader somatosensory system (Seo et al., 2023), it specifically addresses internal feedback, distinct from external sensations like touch, temperature, or pain, which are sensed by cutaneous receptors (Psy and Olman, 2022).

Proprioceptive degradation significantly impairs motor control and balance across clinical populations. Hillier et al. demonstrated that reduced proprioceptive input leads to impaired coordination and postural instability (Hillier et al., 2015). At the same time, Ferlinc et al. reported that age-related proprioceptive decline markedly increases fall risk and reduces mobility (Ferlinc et al., 2019).

Below-knee (transtibial) amputees are particularly affected, as the loss of the foot–ankle complex removes critical mechanoreceptors, disrupting both motor and sensory pathways (Arifin et al., 2014a). This disruption compromises postural control, increases sway, and heightens the risk of falls, particularly during walking perturbations (Olenšek et al., 2021). As a result, transtibial amputees often shift weight to the intact limb and rely heavily on visual and vestibular cues, leading to asymmetrical stance and reduced postural confidence (Arifin et al., 2014b; Vanicek et al., 2009).

Conventional transtibial prostheses lack intrinsic somatosensory feedback (Barberi et al., 2023), forcing users to depend on indirect cues, such as socket pressure or visual monitoring, resulting in compensatory gait deviations (e.g., longer steps, hip hiking, trunk rotation, and knee hyperextension) (Crea et al., 2014; Nguyen et al., 2025). These adaptations reduce gait efficiency, increase energy expenditure, and elevate the risk of long-term musculoskeletal complications, including joint degeneration and low back pain (Teater et al., 2023).

The lack of proprioceptive feedback not only impairs prosthesis control but also affects how it integrates into the user’s body schema, decreasing device acceptance and weakening user confidence (Blanke, 2012; Makin et al., 2017; D’Alonzo and Cipriani, 2012).

Addressing this sensory deficit is therefore crucial, as restoring proprioceptive feedback can enhance both functional performance and user experience (Diaz-Hernandez, 2025).

Emerging evidence highlights the significant benefits of proprioceptive restoration in individuals with transtibial amputations. Improved sensory input enhances a stronger sense of prosthetic ownership and agency, decreases reliance on visual feedback, and promotes more natural and efficient motor control (Diaz-Hernandez, 2025; Preatoni et al., 2021). Experimental interventions that restore plantar or joint-related feedback have been shown to increase stance time and propulsive force on the prosthetic side, improve gait symmetry, and heighten the user’s perception of limb movement (Kim et al., 2023). By re-integrating the prosthesis into the body schema, proprioceptive feedback not only improves functional outcomes but also boosts user confidence and quality of life. Additionally, sensory restoration has been linked to reductions in phantom limb pain by alleviating the cortical sensory mismatch between the residual limb and the prosthetic device (Nguyen et al., 2025).

Despite the recognized importance of proprioception for gait and balance, the literature still lacks a comprehensive, modality-based assessment of sensory feedback systems in transtibial prosthetics. Addressing this gap, the present review examines the question:

How effective are sensory feedback systems in enhancing gait-related outcomes across diverse technologies and rehabilitation contexts?

While prior work (e.g., Diaz-Hernandez, 2025) has examined general feedback technologies or lower-limb prosthetic designs, few reviews compare multiple sensory feedback modalities (invasive, non-invasive) in transtibial amputees with respect to both performance and perceptual outcomes and implementation constraints (Diaz-Hernandez, 2025).

This comprehensive review compares non-invasive modalities (e.g., vibrotactile, electrotactile, Mechanotactile), invasive interventions (e.g., peripheral nerve stimulation, agonist–antagonist myoneural interfaces). The review evaluates key outcomes, including gait symmetry, balance stability, proprioceptive perception, and Phantom Limb Pain (PLP). Furthermore, practical constraints such as latency, training complexity, and interface ergonomics are analyzed. By synthesizing current evidence and identifying critical knowledge gaps, this narrative review provides a foundation for developing a next-generation sensory feedback system that can enhance gait function, confidence, and overall quality of life for individuals with transtibial amputations.

## 2 Methods

A structured narrative review was conducted to synthesize evidence on sensory feedback technologies for transtibial prostheses. Five databases, PubMed, Scopus, Web of Science, IEEE Xplore, and Google Scholar, were searched for articles published between January 2004 and June 2025 using Boolean combinations of the following terms: transtibial amputation, sensory feedback, vibrotactile, electrotactile, Mechanotactile, spinal cord stimulation, peripheral nerve stimulation, myoneural interface, gait, balance, phantom limb pain, embodiment.

Inclusion criteria: (i) original peer-reviewed studies, (ii) adult transtibial or transfemoral amputees, (iii) implementation of invasive or non-invasive sensory feedback interventions, and (iv) reporting of at least one functional or perceptual outcome (e.g., gait symmetry, walking speed, balance stability, phantom limb pain, embodiment).

Exclusion criteria: studies limited to upper-limb prostheses, non-human models, reviews, non-English publications without abstracts, or papers lacking outcome data.

Data extracted included study design, participant characteristics, feedback modality, device configuration, intervention duration, and outcomes. Due to heterogeneity in study designs (ranging from single-case studies to controlled trials), meta-analysis was not feasible. Instead, studies were qualitatively assessed for sample size, intervention duration, ecological validity, and the use of objective metrics. Findings were synthesized thematically by modality and outcome domain to enable cross-comparison of invasive and non-invasive systems.

## 3 Proprioceptive mechanisms and functional significance

The proprioceptive mechanism is regulated by a network of mechanoreceptors, including muscle spindles, Golgi tendon organs (GTOs), and joint capsule receptors. These receptors continuously transmit information regarding muscle length, tension, and joint position to the central nervous system (Zampieri et al., 2020).

Muscle spindles contain 8–20 intrafusal fibers aligned parallel to extrafusal muscle fibers (Macefield and Knellwolf, 2018). Nuclear bag fibers detect changes in muscle stretch velocity (Dolbow and Throckmorton, 2022), while nuclear chain encodes static muscle length (Kröger and Watkins, 2021). Type Ia afferents spiral around both fiber types for rapid dynamic signaling, whereas type II afferents deliver sustained positional feedback (Macefield and Knellwolf, 2018). Gamma motoneuron coactivation maintains spindle sensitivity, optimizing its responsiveness during muscle shortening and movement. This process involves adjusting intrafusal fiber tension in synchrony with extrafusal contraction, thereby preserving responsiveness across length changes (Wilkinson, 2020).

These afferent signals trigger fundamental spinal reflexes, including the monosynaptic stretch reflex, which initiates immediate contraction and antagonist inhibition through Ia input. Additionally, autogenic inhibition occurs when Ib afferents from Golgi tendon organs suppress excessive tension to safeguard muscular structures (Waxenbaum et al., 2024).

Joint capsule receptors, including Ruffini and Pacinian corpuscles, detect joint limits and intra-articular stress (Proske, 2023). They complement muscle spindle feedback, especially near end-range motion and when skin or muscle feedback alone is insufficient (Cordo et al., 2011).

In addition to reflex control, proprioceptive signaling facilitates experience-dependent plasticity (Wu et al., 2021). Repetitive tasks, such as joint-position matching, induce cortical reorganization, thereby enhancing proprioceptive accuracy and motor coordination. These improvements are crucial for rehabilitation and skilled training outcomes (Aman et al., 2015).

Functionally, lower-limb proprioceptive feedback coordinates mediolateral foot placement, step timing, and dynamic balance responses, particularly during gait perturbations (Yiou et al., 2017). Age-related sensory decline or injury-induced sensory loss leads to increased postural sway, impaired reactive control, and reduced gait efficiency by introducing compensatory motor strategies and asymmetric loading (Wingert et al., 2013; Jayasinghe et al., 2020; Henry and Baudry, 2019).

In summary, proprioception is a hierarchical network that includes deep receptors, spinal reflex mechanisms, and cortical adaptation. This network supports adaptive movement. Disruption of this system, caused by aging, injury, or limb loss, leads to significant declines in functions like walking, maintaining posture, and sensorimotor awareness. After transtibial amputation, essential receptors, such as muscle spindles and Golgi tendon organs in the foot–ankle area, are lost. Non-invasive feedback methods, such as vibrotactile or electrotactile systems, aim to mimic position and load signals (Nguyen et al., 2025). Invasive techniques, like the agonist–antagonist myoneural interface, aim to restore proprioceptive signals by mechanically recreating spindle-like input (Srinivasan et al., 2020). Therefore, restoring proprioceptive feedback in prosthetic devices and rehabilitation for transtibial amputees is critical.

## 4 Proprioception loss in transtibial amputees

The amputation of the foot-ankle complex disrupts the fundamental proprioceptive pathways, including muscle spindles, joint receptors, and plantar skin afferents. This deficit necessitates an overreliance on the intact limb and visual-vestibular mechanisms for maintaining balance and gait control.

### 4.1 Intact knee proprioception with asymmetric weight shift

Although the knee capsule remains intact following transtibial amputation, the increased mass and altered center of mass from a prosthesis measurably change movement effort, subtly affecting knee joint position sense despite preserved anatomical integrity (Proske, 2005). Concurrent weight-bearing asymmetries (typically ∼58% on the sound limb vs. ∼42% residual) (Özyürek et al., 2013) amplify mechanical loading, accelerate contralateral joint degeneration, and compound residual thigh muscle weakness (Burger et al., 2005). Collectively, these limitations erode the biomechanical and sensory components that lay the groundwork for impaired balance and increased risk of falls (Horak, 2006).

### 4.2 Elevated falls risk from sensory deficit and motor weakness

The removal of sensory receptors in the plantar surface, muscles, and joints of the amputated limb weakens rapid corrective responses to disturbances (Trotman et al., 2025; Nanivadekar et al., 2023). Without input from distal plantar and joint mechano-receptors, amputees find it harder to make timely gait adjustments and compensate for decreased lower-limb strength (Major et al., 2018). These combined sensory-motor deficits greatly increase the risk of falls and hinder functional stability in daily activities (Petersen et al., 2022). To compensate for these deficiencies, the neuromuscular system must adapt centrally.

### 4.3 Neural adaptation: compensatory cortical control and reflex strategy shifts

Electroencephalography (EEG) and electromyography (EMG) studies have demonstrated that amputees exhibit increased cortical activation (in the alpha, beta, and sigma band frequency ranges) during static balance exercises, even with their eyes open (Khowailed and Khowailed, 2014). This phenomenon suggests an enhanced reliance on central nervous system control due to compromised spinal reflexes and diminished sensory feedback pathways.

The deafferentation of muscle and joint receptors results in altered neuromuscular activation timing and recruitment patterns. Notably, this includes compensatory hip strategies (e.g., elevated hip extensor moments) and a transition from plantar flexor power to proximal muscle control (Curtze et al., 2012). This elevated central control requirement likely contributes to increased cognitive load and fatigue during walking in amputees, as evidenced by significantly higher EEG-derived cognitive activity during ambulation compared to sitting (dry-EEG P3 amplitude notably decreased during walking (Swer et al., 2023).

Given these significant sensory-motor deficits and compensatory cortical strategies, researchers have pursued technological solutions to restore proprioceptive input. These methods generally include invasive and non-invasive prosthetic feedback systems.

## 5 Prosthetic feedback technologies

Modern lower-limb prostheses often fail to provide natural sensory feedback associated with limb movement and ground contact, resulting in unnatural gait patterns and reduced user confidence. Consequently, users perceive the prosthesis as an external entity rather than an extension of their body (Schmitt et al., 2023).

### 5.1 Invasive systems

#### 5.1.1 Transverse intraneural multichannel electrodes (TIME)

The TIME comprises a polyimide thin-film array inserted transversely into peripheral nerves, providing multi-fascicle stimulation via high-density platinum contacts (Boretius et al., 2010). In transtibial cases, TIMEs implanted in the tibial nerve enable precise sensory feedback by selectively activating fascicles (Badia et al., 2011). Animal studies confirmed the chronic biocompatibility of TIME-3, showing no demyelination after 2 months (Badia et al., 2011). In contrast, human trials in transradial amputees demonstrated stable, focal sensation (Strauss et al., 2019). However, the technique is invasive and technically demanding, and the long-term risk of foreign-body reactions and electrode migration remains a significant concern (Boretius et al., 2010; Badia et al., 2011).

#### 5.1.2 Agonist-antagonist myoneural interface (AMI)

An alternative approach is the Agonist-Antagonist Myoneural Interface (AMI). A surgical procedure that links pairs of residual muscles in an agonist–antagonist configuration, preserving natural proprioceptive feedback by stretching one muscle when its counterpart contracts (Srinivasan et al., 2020; Herr and Carty, 2021). Clinically applied in transtibial amputees, AMI constructs have demonstrated enhanced control over prosthetic joints, dynamic reflexive behaviors during stair walking, maintenance of muscle bulk, and improved phantom joint perception (Srinivasan et al., 2020; Srinivasan et al., 2017). While AMI offers compelling improvements in neuromechanical integration and embodiment, it imposes surgical complexities, including extended operative times and precise tissue handling, and may be impractical for broader patient populations without healthy residual musculature (Tang et al., 2024).

#### 5.1.3 The composite flat interface nerve electrode (C-FINE)

The C-FINE is an epineural wrap with a PEEK core and silicone layers that reshape nerves to increase electrode contact while preserving flexibility (Freeberg et al., 2017; Yildiz et al., 2020). In transtibial applications, C-FINEs installed around sciatic/tibial nerves provide high contact density (e.g., 16 channels) for selective stimulation (Charkhkar et al., 2018). Clinically, they offer enhanced long-term stability and reduced cuff bulk, but their invasiveness, need for percutaneous leads, and early postoperative threshold variability limit broad use (Freeberg et al., 2020).

Although invasive neural interfaces can achieve a high level of homology (matching the sensation type) and somatotopy (matching the sensation’s spatial location), thereby improving prosthesis acceptance and embodiment (Di Pino et al., 2020), they face several limitations. These include limited long-term biocompatibility, the risk of scar tissue formation around electrodes, and potential infection or nerve damage from surgical implantation (Valle et al., 2022). Additionally, these systems are costly and technically complex, which currently limits their clinical use (Adewole et al., 2016). For example, intraneural electrode implantation, although precise, requires microsurgical techniques and may experience signal degradation over time due to fibrotic encapsulation, often necessitating surgical revision (Gori et al., 2021). Their direct neural access provides very low-latency transmission, which is crucial for stumble recovery during gait. However, the significant surgical burden still limits scalability and widespread use in lower-limb prosthetic rehabilitation.

### 5.2 Non-invasive systems

Non-invasive somatosensory feedback systems have gained significant attention (Svensson et al., 2017). These solutions externally stimulate the skin, avoiding surgical risks and enhancing practicality for daily use (Moshayedi et al., 2024).

Three primary non-invasive feedback modalities are being explored.

#### 5.2.1 Electro-tactile feedback

Electro-tactile feedback uses transcutaneous electrical nerve stimulation to deliver pressure, movement, or tactile sensations through the skin in a lightweight setup (Manoharan and Park, 2023). In lower-limb applications, electrodes on the stump or thigh encode foot pressure or joint angles to evoke tactile percepts that enhance proprioceptive awareness and reduce phantom pain (Basla et al., 2021). Its advantages include precise parameter tuning and quick response, but electric fields may cause discomfort, cognitive distraction in some users, and interference with EMG-based control (Bensmaia et al., 2020; See et al., 2022).

#### 5.2.2 Mechanotactile feedback

Mechanotactile feedback uses localized pressure, often via tactors, to deliver intuitive, modality-matched sensations by directly mirroring contact at the prosthesis onto the skin. This method naturally conveys grasp force, the onset and offset of touch, and contact location in a perceptually consistent way (Morita et al., 2016; Casini et al., 2015). Although Mechanotactile systems can provide naturalistic feedback, they are usually bulkier and consume more energy than vibrotactile or electrotactile options (Bensmaia et al., 2020). Nonetheless, early wearable Mechanotactile sleeves have shown higher grasp success and decreased muscle effort in prosthetic hand users (Borkowska et al., 2022). While most Mechanotactile research has concentrated on upper-limb prostheses (Shehata et al., 2020; Schoepp et al., 2018), the core principle of mapping contact forces directly onto the skin is equally applicable for lower-limb gait support, such as conveying plantar pressure or stance phase cues in transtibial prostheses, where accurate load and timing information are essential for safe ambulation (Martini et al., 2020).

#### 5.2.3 Vibrotactile feedback

Vibrotactile feedback uses small vibration motors applied to the skin to stimulate Pacinian corpuscles, providing artificial sensation through vibration (Stephens-Fripp et al., 2018). In transtibial prostheses, it has been used in three main ways: (1) center-of-pressure cues, where sensorized insoles activate skin-mounted tactors to improve gait symmetry and reduce visual load (Rusaw et al., 2012; Plauche et al., 2016); (2) underfoot object localization (Wan et al., 2016), using thigh arrays to help users identify foot placement during stairs or uneven terrain, increasing placement accuracy by up to 17% (Rokhmanova and Rombokas, 2019); and (3) joint angle encoding, where thigh-mounted vibration motors signal ankle or knee angles during myoelectric control, cutting position error in virtual tasks in half (Marayong et al., 2014; Chen et al., 2016).

Among non-invasive systems, vibrotactile feedback is of particular interest because it is both affordable and does not interfere with the situational awareness of the user (Wu et al., 2025; Shull et al., 2014). Clinically, vibrotactile systems avoid skin irritation common with electrotactile stimulation (Ray et al., 2021), and are much lighter and less complex than Mechanotactile arrays (Bensmaia et al., 2020). Their limitations include lower spatial resolution and potential habituation over time (Colan et al., 2024).

In summary, non-invasive feedback modalities provide practical, lower-risk, and user-friendly means to restore sensory information, thereby enhancing embodiment, gait efficiency, balance, and functionality in users of lower-limb prostheses according to many studies. They serve as a critical point toward more intuitive and natural prosthetic control. Furthermore, Non-invasive stimulation induced similar improvements in dual motor and cognitive tasks compared to neural feedback (Chee et al., 2022a).

## 6 Literature synthesis and comparative analysis

This section reviews recent studies that assess sensory feedback in individuals with transtibial amputations. Both invasive and non-invasive strategies are examined. Invasive approaches include intraneural stimulation (Schmitt et al., 2023) and the agonist–antagonist myoneural interface (Song et al., 2024). Non-invasive approaches include vibrotactile (Rokhmanova and Rombokas, 2019; Valette et al., 2025; Westlake and Culham, 2007), electrotactile (Chee et al., 2022a; Dietrich et al., 2018; Demofonti et al., 2025), and Mechanotactile feedback (Bachini et al., 2022), as shown in Table 1. Findings are organized around key outcomes: gait symmetry, balance and stability, and phantom limb pain, as shown in Table 2. This structure enables direct comparison of how different methods contribute to functional and perceptual improvements.

**TABLE 1:** Study characteristics.

Author (Year)	N/Level	Setting	Device/Modality	Training dose
Bachini et al. (2022)	1 TFA	Treadmill, lab	Mechanical feedback via socket (patch + stiffness)	Single session
Rokhmanova and Romboka (2019)	2 TTA +10 controls	Lab (stair stepping)	Vibrotactile (thigh) + sensorized insole	∼1–1.5 h evaluation (120 trials)
Escamilla-Núñez et al. (2023)	3 TFA/2 TTA	Lab (treadmill)	Vibrotactile for STSR correction	∼1–2 h, 16 trials
Kalff et al. (2024)	11 mixed	Home + lab	Gait-synchronized vibrotactile (thigh)	∼61 days unsupervised home use
Valette et al. (2025)	2 TTA/1 TFA +10 controls	Lab + walking test	Vibrotactile + sensorized insole	∼4 h single session
Dietrich et al. (2018)	14 TTA	Indoor/outdoor walking	Electrocutaneous + sensorized insole	10 days (2 × 2 h/day)
Chee et al. (2022a)	3 TFA	Lab (dual task and perception)	Electrocutaneous (pressure/knee flexion)	Single session
Basla et al. (2021)	3 TFA +3 controls	Lab (treadmill)	Electrocutaneous + sensorized insole + IMU	10 min calibration + task practice
Valette et al. (2023)	3 TTA/3 TFA +11 controls	Lab (treadmill)	Electrocutaneous	∼2.5 h single session
Demofonti et al. (2025)	Phase 1: 13 mixed (6 TTA, 7 TFA); phase 2: 2 (S1: TTA, S7: TFA)	Lab (treadmill)	Electrocutaneous (nerve electrodes)	4 weeks treadmill training
Schmitt et al. (2023)	1 TTA (case)	Home/community	Implanted peripheral nerve + insole sensors	∼31 weeks daily use
Song et al. (2024)	7 TTA (AMI) + 4 controls	Lab + real terrains	AMI with implanted sensors	Single post-rehab evaluation

TFA, transfemoral amputation; TTA, transtibial amputation; STSR, stance time symmetry ratio; IMU, inertial measurement unit; AMI, agonist–antagonist myoneural interface; SCS, spinal cord stimulation.

**TABLE 2:** Outcomes by modality.

Modality/Study	Gait symmetry/Speed	Balance/Stability	PLP
Mechanical (Bachini et al., 2022)	↑ heel/toe angles; ↓ double support	Improved calf perception → better gait control	Not reported; non-painful phantom sensations
Vibrotactile (Rokhmanova and Rombokas, 2019)	Not assessed	↑ foot placement accuracy (+15–20%)	Not assessed
Vibrotactile (Escamilla-Núñez et al., 2023)	↑ gait symmetry, speed, stride length	Not assessed	Not assessed
Vibrotactile (Kalff et al., 2024)	↑ 10 m walk speed in 45%	↓ TUG time; FSST improved in 36%	Not reported
Vibrotactile (Valette et al., 2025)	SI ↑ (TT1: 58→75%; TT2: 65→75%; TF1: 56→74%)	↑ confidence; ↓ floor fixation	Not assessed
Electrocutaneous (Dietrich et al., 2018)	↑ walking distance	↑ stability and confidence	PLP ↓ 2.3→1.9
Electrocutaneous (Chee et al., 2022b)	↓ metabolic cost; more physiological gait	↑ stability and confidence	Not reported
Electrocutaneous (Basla et al., 2021)	SI ↑ 16%–48%; stance/swing ratio ↑ 3%–10%	Balance ↑ 32.7→82.8%	Not assessed
Electrocutaneous (Valette et al., 2023)	Walking speed: amputees 0.73 m/s; controls 1.02 m/s	Sensory discrimination ↓	Not assessed
TENS/SCS (Demofonti et al., 2025)	↑ step length and stance symmetry; ↑ walking speed and vGRF	Weight distribution closer to physiological	S1: 6→0; S7: no pain
Implanted nerve (Schmitt et al., 2023)	More natural gait; ↓ heel stomping	↑ automatic stability on uneven terrain	High stim → unpleasant phantom sensations
AMI (Song et al., 2024)	↑ 41% max walking speed; more symmetric step and ankle power	↑ slope/stairs/perturbation performance	Not reported

SI, symmetry index; TFA, transfemoral amputation; TTA, transtibial amputation; AMI, agonist–antagonist myoneural interface; SCS, spinal cord stimulation; ↑/↓ indicate improvement or reduction; TUG, timed up and go; FSST, four square step test; GRF, ground reaction force; SOT, sensory organization test; FGA, functional gait assessment.

**FIGURE 1:**
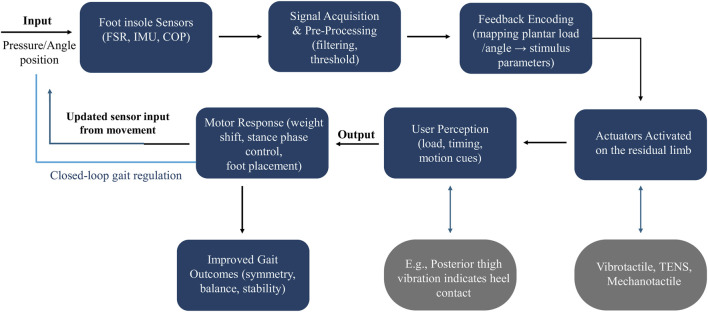
Systems-level conceptual framework, illustrating how sensory feedback flows from prosthetic foot sensors through signal processing and feedback encoding stages to actuators on the residual limb.

To clarify how sensory feedback contributes to gait improvements across modalities, Figure 1 presents a framework of a closed-loop system. Sensory information from sensors (e.g., FSRs, IMUs, COP sensors) is processed and encoded into tactile or electrotactile stimuli. These stimuli are delivered through actuators mounted on the residual limb, producing perceptual cues about load, timing, or joint motion. Users integrate these cues into motor responses such as stance stabilization, improved weight shifting, or more accurate foot placement. These adapted responses contribute to improved functional outcomes, including gait symmetry, balance, and reduced energetic and cognitive demand.

## 7 Discussion

### 7.1 Interpretations, gaps, engineering implications

Loss of somatosensory feedback remains a primary barrier to the restoration of natural gait in transtibial amputees (Chee et al., 2022b). The absence of plantar pressure and proprioceptive cues contributes to gait asymmetry (Arifin et al., 2014b), increased metabolic cost (Chee et al., 2022b), elevated fall risk (Kim et al., 2023), and greater reliance on visual input (Fuchs et al., 2021). Sensory neuroprostheses, both invasive and non-invasive, can restore portions of these feedback channels, improve stability and symmetry, and reduce cognitive load during dual-task walking (Tian et al., 2025).

Restoring proprioceptive input through feedback systems, both invasive and non-invasive, engages multiple neurophysiological mechanisms. Feedback systems reintroduce afferent signals, supporting reflexive responses and reducing reliance on vision (Chee et al., 2022b; Fuchs et al., 2021; Tian et al., 2025). At the cortical level, repeated sensory input promotes cortical plasticity, remapping deafferented sensorimotor areas and enhancing integration of prosthetic signals, which also helps reduce phantom limb pain (Basla et al., 2021; He et al., 2022). This adaptive reorganization supports motor learning, allowing users to refine timing, force, and coordination, resulting in more natural gait patterns (Li et al., 2023). Moreover, reinstating somatosensory cues enhances embodiment, increasing ownership of the prosthesis and leading to a more confident and efficient gait (Di Pino et al., 2020; Tian et al., 2025). Collectively, these neurophysiological mechanisms underline the potential of sensory feedback systems to restore both perception and function in transtibial amputees.

Prior reviews highlight gaps in comparing modalities and addressing real-world applicability. Diaz-Hernandez et al. (2025) surveyed sensory feedback modes and neural interface innovations but provided limited coverage of implementation constraints such as latency, training burden, and usability (Diaz-Hernandez, 2025). Manz et al. (2022) cataloged user needs, including stability, comfort, walking versatility, and confidence, but lacked technical comparison across modalities (Manz et al., 2022). The present review integrates objective functional outcomes and PLP measures, evaluates deployment and ergonomic constraints, and provides a framework for assessing feasibility in transtibial sensory feedback systems.

A comparison of invasive, electrotactile, mechanotactile, and vibrotactile modalities reveals distinct trade-offs. Invasive neural feedback offers high spatial and temporal fidelity, often yielding somatotopic sensations in the phantom foot or ankle that enhance embodiment and performance under demanding conditions (Di Pino et al., 2020); however, these benefits involve surgical risks, long-term electrode instability, and regulatory burdens (Valle et al., 2022; Gori et al., 2021). Non-invasive electrotactile arrays allow flexible encoding via adjustable amplitude, pulse width, and spatial mapping but may suffer perceptual degradation during walking, discomfort from skin movement or humidity, and higher cognitive remapping demands (Basla et al., 2021; Bensmaia et al., 2020). Mechanotactile systems provide more natural mechanical cues but are less frequently tested in transtibial prosthetic settings due to bulk, energy demands, and integration challenges (Bensmaia et al., 2020). These functional differences are complemented by ethical and practical considerations relevant for clinical translation. Non-invasive systems generally provide broader patient accessibility, lower cost, and moderate training requirements, whereas invasive systems, despite higher fidelity, involve greater cost, and more extensive training and long-term management (Gori et al., 2021; Chee et al., 2022a).

Among non-invasive options, vibrotactile feedback has recently demonstrated the greatest feasibility in real-world settings, pointing to the value of further research in this area. The hardware for vibrotactile feedback is relatively simple, compared to other non-invasive methods, consisting of multiple sensors and vibration motors. It is easier to implement than electro-tactile techniques and avoids the bulk and energy usage associated with mechanotactile systems. Compared to invasive options, vibrotactile feedback offers nearly the same functional benefits while being more affordable and eliminating surgical risks (Chee et al., 2022a).

Based on existing studies, the Vibrotactile Foot Sensor System (VTFS) (Kalff et al., 2024) used four pressure sensors embedded in a sock beneath the prosthetic foot, paired with four vibration motors mounted on the thigh. After approximately 61.5 days of home use, VTFS significantly improved gait stability according to the Timed Up and Go test (p = 0.042). Five out of 11 (about 45%) participants showed a clinically meaningful increase in walking speed on the 10-m Walk Test. Improvements in coordination, measured by the Four-Square Step Test (FSST), were observed in 36% of participants, although these did not reach statistical significance, but showed upward trends. Only 9% achieved clinically relevant gains in endurance on the 2-Minute Walk Test. Importantly, user feedback was positive: 63% rated their VTFS experience as “good” or better, and 45% expressed a desire to continue using the device.

Limitations of vibrotactile feedback studies include small sample sizes (usually 15 or fewer participants) (Kalff et al., 2024; Dietrich et al., 2018), limited diversity in study populations, short intervention durations, and underrepresentation of real-world scenarios such as uneven terrain, stairs, perturbations, and dual-task walking (Rokhmanova and Rombokas, 2019; Escamilla-Nunez et al., 2023; Valette et al., 2025). Some studies, including VTFS, did not show statistically significant improvements at the group level despite considerable individual gains (Kalff et al., 2024). From an engineering perspective, device features such as sensor–actuator latency, vibration frequency, actuator placement, damping effects from socket or liner materials, and durability against sweat or movement artifacts are often inconsistently reported (Bensmaia et al., 2020; Basla et al., 2021; Valette et al., 2023). Embodiment, comfort, fatigue, energy consumption, and battery life are also rarely assessed in a standardized manner (Chee et al., 2022a; Shah et al., 2018; Pomplun et al., 2022).

Inter-individual differences further complicate interpretation and generalization. Factors such as skin sensitivity, limb shape, age, and other health conditions interact with prosthesis-related factors like amputation level, residual limb length, socket design, and user adaptation, influencing sensory perception and feedback responses (Pomplun et al., 2022; Valette et al., 2023). For example, transfemoral amputees tend to lose more mechanoreceptors than transtibial amputees, but compensatory strategies via the hip joint and stump–socket pressure cues may lessen these deficits (Latanioti et al., 2013; Rabuffetti et al., 2005). Transtibial amputees retain more structural elements but still show reduced sensory thresholds and balance issues (Arifin et al., 2014a; Olenšek et al., 2021). Residual limb length, socket fit, suspension type, soft tissue, and prosthesis experience further impact on feedback reliability (Baum et al., 2008; Dunn et al., 2024).

Future work should address both technical and user-centered gaps: (i) larger and more diverse cohorts; (ii) long-term trials ≥6–12 months in real-world settings (stairs, perturbations, community walking); (iii) direct comparisons across modalities under matched task and dual-task conditions; (iv) standardized reporting of vibration frequency, spatial channels, actuator placement, and latency; (v) systematic measurement of cognitive load, embodiment, comfort, fatigue, energy use, and battery life; and (vi) robust socket integration with individual adjustability and calibration in dynamic environments.

### 7.2 Limitations of the review

In conducting a comprehensive synthesis of sensory feedback technologies for transtibial amputees, this review has several limitations. First, the evidence base is potentially influenced by publication bias, as studies reporting positive or significant outcomes are more likely to be published, whereas studies with null or negative results may be underrepresented. Second, many of the included studies had small sample sizes, often fewer than 15 participants, which limited statistical power and reduced generalizability to the broader amputee population. Third, there is considerable heterogeneity in study designs, including differences in feedback modalities, prosthetic components, intervention duration, outcome measures, and assessment methods. This variability precluded meta-analytic synthesis and necessitated a qualitative approach. Finally, although a structured search strategy across multiple databases was implemented, relevant studies may have been missed due to inconsistent indexing or variations in terminology.

## 8 Conclusion

This review underscores the central role of somatosensory feedback in restoring natural gait for transtibial amputees. Evidence indicates that invasive neural interfaces provide unmatched fidelity and embodiment but are limited by surgical risks, hardware stability issues, and scalability constraints. Non-invasive modalities, particularly vibrotactile systems, demonstrate meaningful improvements in gait symmetry, balance, and user confidence, with favorable safety and usability profiles. However, their effectiveness is often constrained by small sample sizes, short intervention durations, and laboratory-based conditions.

Vibrotactile feedback currently offers the most practical near-term solution for clinical deployment, whereas invasive systems remain the benchmark for high-fidelity performance in specialized settings. Realizing the translational potential of sensory neuroprostheses requires future research to prioritize larger and more diverse participant cohorts, long-term real-world trials, and standardized reporting of both engineering metrics (e.g., latency, energy consumption, actuator placement) and clinical outcomes (e.g., gait symmetry, metabolic cost, phantom limb pain, embodiment, cognitive load).

Despite these advances, several limitations persist. Heterogeneity in study designs, inconsistent reporting of device parameters, and inter-individual variability restrict generalizability. Addressing these gaps through robust socket integration, extended real-world evaluations, and harmonized outcome measures will be essential for developing next-generation prosthetic feedback systems that are technologically advanced, clinically viable, and capable of restoring confident, natural mobility in daily life.
